# A 24 GHz-Optimized Up-Conversion Mixer for Beyond-5G: A Combined Com_GAPSO_ and Im_GKAN_ Approach

**DOI:** 10.3390/mi17070794

**Published:** 2026-06-29

**Authors:** Unal Aras, Tahesin Samira Delwar, Khizra Tariq, Mangal Singh, Sayak Mukhopadhyay, Yangwon Lee, Jee-Youl Ryu

**Affiliations:** 1 Department of Smart Robot Convergence and Application Engineering, Pukyong National University, Busan 48513, Republic of Korea; aras2120@pukyong.ac.kr (U.A.); samira95@ks.ac.kr (T.S.D.); khizra_tariq@pukyong.ac.kr (K.T.); 2 Department of Global IT Engineering, Kyungsung University, Busan 48513, Republic of Korea; 3 Department of Electronics and Telecommunication Engineering, Symbiosis Institute of Technology, Pune Campus, Symbiosis International, Deemed University, Pune 412115, India; mangal.etce@gmail.com (M.S.); sayakpm3@gmail.com (S.M.); 4 Department of Spatial Engineering, Pukyong National University, Busan 48513, Republic of Korea

**Keywords:** 5G, genetic algorithms, improved-Kolmogorov–Arnold networks, particle swarm optimization, up conversion mixer

## Abstract

An optimal CMOS up-conversion mixer is designed using a novel combination of genetic algorithms and particle swarm optimization (Com_GAPSO_) and improved-Kolmogorov–Arnold networks (Im_GKAN_) for 5G communication. The proposed Im_GKAN_, trained with Com_GAPSO_, enhances optimization through social interactions and private cognition through social interactions. The proposed hybrid approach enables accurate parameter determination due to the effective modeling and compensation of nonlinearities in the up-conversion mixer. The proposed optimized mixer incorporates an enhanced linearity boosting technique (LBT) along with a tunable capacitive feedback common-source (TCF-CS) structure. This combination effectively suppresses third-order nonlinear distortion while compensating for parasitic capacitances to improve gain performance and enhance circuit stability. The proposed design achieves a peak conversion gain (CG) of approximately 4.2 dB near 24 GHz. In terms of isolation characteristics, the LO-IF isolation reaches about −44 dB. Additionally, the RF-IF isolation is around −30 dB, ensuring minimal undesired coupling between the input and output paths, while the LO-RF isolation is maintained near −39 dB. The optimized mixer exhibits an output 1 dB compression point (OP_1_dB) of 5.1 dBm and an input 1 dB compression point (IP_1_dB) of −1.1 dBm. The RF port shows a return loss of approximately −24 dB near 24 GHz. The LO port exhibits a return loss in the range of −3 to −5 dB, with improved matching observed over the operating band. Meanwhile, the IF port demonstrates strong matching at lower frequencies, with return loss values dropping below −20 dB. Furthermore, the measured optimized design achieves a minimum noise figure (NF) of approximately 3.8 dB at 24 GHz.

## 1. Introduction

During the past few years, wireless communication systems have evolved rapidly, so high data rates, low latency, and increased capacity have become increasingly important, particularly in Beyond-5G (B-5G) communications. This is where B-5G systems and capabilities come into play, as they need to meet these demands while also delivering performance enhancements that surpass the capabilities of existing LTE and 5G networks [1] A key component of B-5G communication is the development of efficient, low-power, and highly linear RF circuits. As part of this communication chain, up-conversion mixers play an active role, directly impacting linearity, gain, and overall efficiency.

To overcome the challenges of transmitter design, it is essential to be equipped with an up-conversion mixer that can be used to achieve high linearity and high gain while minimizing the need for additional amplification stages, which can degrade linearity [2]. Additionally, for advanced communication systems, up-conversion mixers operating at frequencies between 20 and 30 GHz are particularly useful, as they offer high data throughput and wide bandwidth [3].

### 1.1. A. Motivational Perceptions into the Com_GAPSO_–Im_GKAN_ Mixer Design

**Figure 1:**
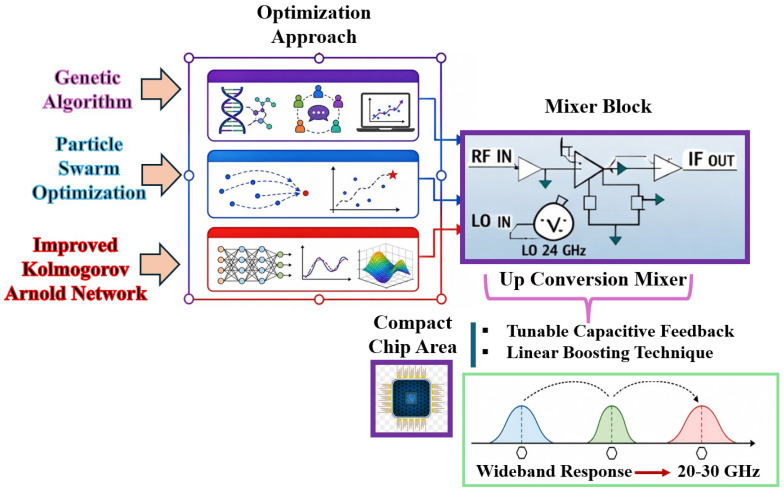
Overview of the proposed 24 GHz optimized up-conversion mixer.

A new approach is presented by Im_GKAN_, which integrates GAs along with PSOs to address the limitations of traditional up-conversion mixer designs by integrating their strengths and weaknesses in the design process. There are two main mechanisms used to achieve convergence in local search spaces: GA, which has robust exploration capability for global optimization, and PSO which has the capability for solving local problems [4,5,6]. Thus, it is possible to design highly efficient RF circuits that meet the performance requirements for B-5G. Figure 1 presents an overview of the proposed 24 GHz optimized up-conversion mixer.

#### B. Related Literature

Mixers used in RF systems are broadly classified into passive and active types, each offering distinct trade-offs in performance. Passive mixers rely on switching mechanisms driven by the local oscillator (LO) to perform frequency translation, resulting in inherently low power consumption, high linearity, and favorable noise characteristics [7,8]. However, they suffer from conversion loss and typically demand relatively high LO drive levels. In contrast, active mixers provide conversion gain, improved port isolation, and reduced LO power requirements, making them more suitable for integrated circuit implementations despite their increased power consumption [9,10].

Prior studies have explored various CMOS mixer topologies to enhance performance metrics such as noise figure (NF), conversion gain (CG), and linearity. For instance, designs in [11,12] focused on minimizing noise and improving NF, whereas others [13,14,15] achieved gains in specific parameters at the expense of linearity or isolation. Additionally, alternative configurations applying RF and LO signals at different transistor terminals [16] and techniques such as derivative superposition and active baluns [17,18] have been introduced, though often with penalties in power efficiency.

To further improve mixer performance, numerous linearization approaches have been proposed, particularly in Gilbert-cell-based architectures, which are widely adopted due to their superior isolation and gain characteristics [19,20]. Enhancements such as dual transconductance boosting [21], cascode folding [22], source degeneration [23], class-AB transconductance stages [24], and harmonic suppression techniques [25] have demonstrated notable improvements. However, these methods often increase circuit complexity or degrade gain.

Although prior works have reported mixers operating in the millimeter-wave range (15–35 GHz), most designs focus on optimizing a single parameter, such as gain or linearity, rather than achieving a balanced improvement. Only limited studies, such as [26], have addressed both high conversion gain and linearity simultaneously at 24 GHz; yet these lack comprehensive technical analysis and are primarily targeted at automotive radar applications. In contrast, the growing demand for Beyond-5G systems necessitates mixer designs that simultaneously achieve high linearity, gain, and power efficiency at 24 GHz frequencies.

In addition, Yang et al. [27] proposed a 24–30 GHz mixer employing a novel linearization technique to achieve enhanced linearity without significantly increasing power consumption. Bae and Han [28] introduced a wideband CMOS mixer operating from 24 to 40 GHz for 5G FR2 applications, demonstrating improved bandwidth and gain characteristics. Lin and Lan [29] reported a high-conversion-gain mixer covering 12.4–32 GHz for 28 GHz 5G New Radio systems. Furthermore, Wang and Wen [30] presented a broadband 30–60 GHz CMOS mixer with low LO drive requirements, targeting next-generation millimeter-wave transceivers. Collectively, these studies demonstrate the increasing importance of intelligent optimization frameworks for achieving multi-objective performance enhancement in millimeter-wave mixers intended for Beyond-5G and future 6G transceivers.

The remainder of this paper is organized as follows. Section 2 presents the design of the proposed up-conversion mixer system. Section 3 describes the up-conversion mixer optimization process using the Com_GAPSO_–Im_GKAN_ approach. The results and discussion are provided in Section 4.

### 1.2. Main Contributions

The novelty of this work lies in its optimization methodology and its impact on circuit performance. We use a Com_GAPSO_-backed Im_GKAN_ for RF parameter optimization. This approach captures complex nonlinear relationships between design variables and performance metrics more effectively than conventional tuning. It integrates global search capabilities with learning-based nonlinear modeling. The Im_GKAN_ model also enables implicit nonlinear compensation during parameter selection.

1. A new hybrid optimization approach, Com_GAPSO_–Im_GKAN_, is proposed by integrating GA and PSO to exploit both evolutionary search capability and swarm intelligence for efficient parameter optimization.
2. The proposed Im_GKAN_ accurately models and compensates for nonlinear behavior in the up-conversion mixer, enabling precise circuit parameter tuning and improved performance.
3. An advanced LBT techniques combined with a TCF-CS structure is introduced to suppress third-order nonlinear distortion and mitigate parasitic capacitances.
4. The measured optimized mixer achieves good RF performance, including higher CG (4.2 dB), improved linearity OP_1_dB = 5.1 dBm, IP_1_dB = −1.1 dBm, and reduced NF (3.8 dB) at 24 GHz.
5. The proposed design demonstrates reduced power consumption (3.4 mW at 1.2 V), which is suitable for beyond-5G applications.

## 2. Circuit Architecture and Implementation

**Figure 2:**
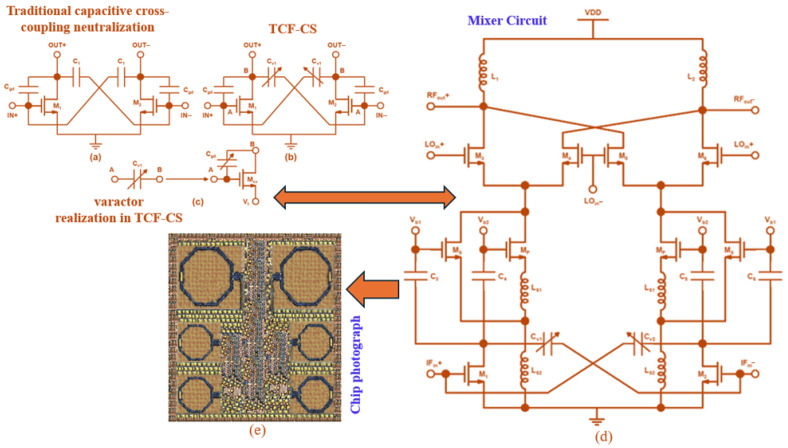
Circuit Details (**a**) Traditional capacitive neutralization, (**b**) Proposed TCF-CS structure, (**c**) Varactor implementation, (**d**) Proposed mixer, (**e**) Chip micrograph.

The proposed up-conversion mixer, illustrated in Figure 2, converts a 2.4 GHz IF signal to a 24 GHz RF signal. The IF signal is first amplified by the G_m_ stage, which consists of a tunable capacitive feedback common-source (TCF-CS) structure and linearity boosting technique (LBT) block. The TCF-CS stage uses transistors M_1_ and M_2_ along with varactors (C_v1_, C_v2_), whose capacitance is controlled by a tuning voltage (V_t_). These varactors cancel the parasitic gate-drain capacitance (C_gd_) through capacitive neutralization, improving gain and stability. The tuning transistor operates in the subthreshold region to minimize power consumption.

The LBT stage enhances linearity by combining primary (M_p_) and secondary (M_s_) transistors with source degeneration inductors (L_s1_, L_s2_). The primary transistor operates in strong inversion, while the secondary transistor operates in moderate inversion, reducing gate noise and improving linearity compared to conventional derivative superposition techniques. The amplified signal is then fed into the switching stage (M_3_–M_6_), where a 21.6 GHz LO signal is applied to perform frequency up-conversion, generating a 24 GHz RF output. Finally, the RF output is delivered through a push–pull buffer (using PMOS M_pb_ and NMOS M_nb_ with a feedback resistor (Rf) to achieve proper 50 Ω impedance matching. Overall, the combination of TCF-CS for gain enhancement and LBT for linearity improvement results in a high-performance, low-noise up-conversion mixer. Table 1 shows the proposed mixer simulation parameters.

### 2.1. Optimization Process Using Com_GAPSO_–Im_GKAN_ Approach

**Figure 3:**
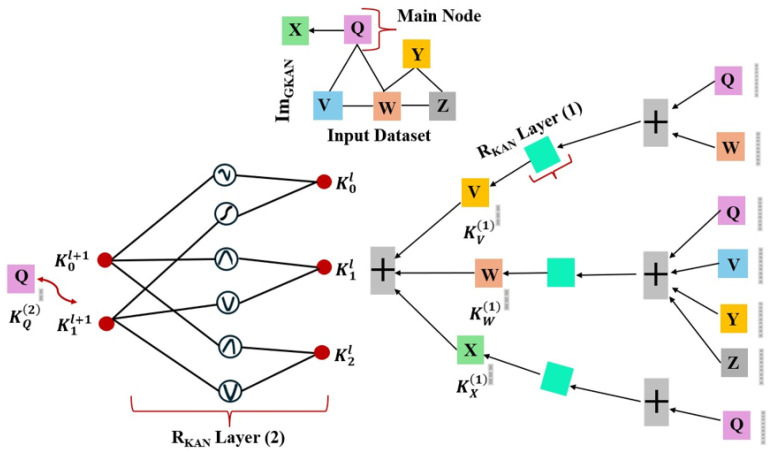
Proposed Im_GKAN_ Approach.

Our present implementation of improved GKAN incorporates KAN layers: architecture 1, which applies the learnable functions to aggregation features after they have been reconstructed. Figure 3 shows the proposed architecture of the Im_GKAN_.

#### 2.1.1. Proposed Im_GKAN_ Architectures

In this architecture, the embedding of nodes at layer *ℓ* + 1 are basically generated by passing the aggregated (e.g., summation) node embedding at layer *ℓ* through KAN layer (*ℓ*), which is represented in Equations (1) and (2).

(1) $$H_{\mathrm{Archit}.\;1}^{\left(\ell +1\right)}=\mathrm{KANlayer}\left(AH_{\mathrm{Archit}.\;1}^{\left(\ell \right)}\right)$$

(2) $$H_{\mathrm{Archit}.\;1}^{\left(0\right)}=X$$

The KAN layer, although appearing simple in Equation (2), presents challenges for optimization. Here, we find the following:

1. Residual Activation Functions: The activation function *ϕ*(x) combines a basis function b(x), reminiscent of residual connections, and a spline function, as presented in Equation (3):

(3) $$\phi \left(x\right)=w_{b}\;b\left(x\right)+w_{s}\;\mathrm{spline}\left(x\right)$$

The basis function is typically defined as in Equation (4):

(4) $$b\left(x\right)=\mathrm{silu}\left(x\right)=\frac{x}{1+e^{-x}}$$

The spline component is expressed as a weighted sum of B-splines as in Equation (5):

(5) $$\mathrm{spline}\left(x\right)=\sum_{i}c_{i}B_{i}\left(x\right)$$

where c_i_ are coefficients that can be adjusted during training. Notably, w_b_ and w_s_ could be absorbed into b(x) and spline(x) but are kept separate to fine-tune the function’s amplitude.

2. *InitializationScales*: The activation functions are initialized such that w_s_ = 1 and spline (x) ≈ x. w_b_ is set based on the Xavier initialization scheme, traditionally used for initializing layers in the MLP as in Equation (6):

(6) $$R_{\mathrm{MLP}}=\left(W_{3}\circ \sigma_{2}\circ W_{2}\circ \sigma_{1}\circ W_{1}\right)\left(x\right)$$

3. Dynamic Spline Grid Updates: The spline grids are updated dynamically based on the input activations. This modification caters to the inherent bounded nature of spline functions, accommodating the evolutionary nature of activation values during training.

Considering L number of layers for these architectures, then the forward model is presented as Z = soft max(H(L) Archit. 1) as shown in Equations (7) and (8):

(7) $$Z=\mathrm{soft}\;\max\left(H^{\left(L\right)}\right)$$

(8) $$R_{\mathrm{KAN}}\left(x\right)=\left(\phi_{3}\circ \phi_{2}\circ \phi_{1}\right)\left(x\right)$$

**Figure 4:**
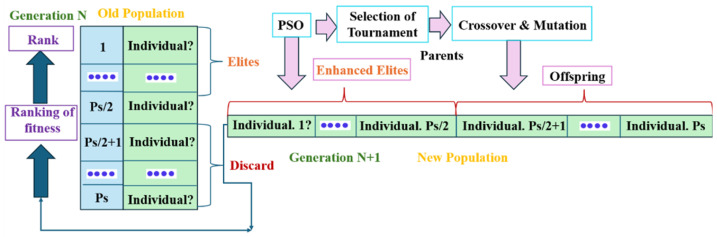
Flow Chart (Com_GAPSO_).

The mixer is represented as a graph G = (V,E), where nodes V denote circuit elements (transistors, passive components, RF/LO/IF ports) and edges E represent signal flow and nonlinear interactions. In Figure 4, we can see the proposed Im_GKAN_ approach configuration.

Each node is associated with a feature vector containing x_i_ device parameters, bias conditions, and parasitic effects. The learning objective is to approximate the nonlinear mapping in Equations (9)–(12):

(9) $$y=f_{\mathrm{RF}}\left(X\right)$$

The Im_GKAN_ layer updates node representations as:

(10) $$h_{i}^{\left(l+1\right)}=\sum_{j\in N(i)}\phi_{l}\left(W_{l}h_{j}^{\left(l\right)}\right)$$

The final prediction is:

(11) $$\hat{y}=\Phi_{3}\circ \Phi_{2}\circ \Phi_{1}\left(X\right)$$

Training data are generated via circuit simulations under parameter sweeps, and the model is trained using:

(12) $$L=∥y-\hat{y}{∥}^{2}$$

Unlike conventional surrogate models, the proposed Im_GKAN_ leverages both graph topology and learnable nonlinear basis functions to effectively capture strong nonlinearities and component-level interactions in up-conversion mixers. The trained model is integrated with the Com_GAPSO_ optimization framework to enable efficient exploration of the design space and accurate determination of optimal circuit parameters.

#### 2.1.2. Optimization Process Im_GKAN_ Using Com_GAPSO_

The proposed Com_GAPSO_ combines GA and PSO to improve optimization performance. By integrating GA’s evolutionary mechanisms with PSO’s social learning strategy, the hybrid approach achieves better results than using either method independently.

1. Initialization: In Com_GAPSO_, both GA and PSO operate on a shared population. Initially, individuals are randomly generated and can be viewed as chromosomes (GA) or particles (PSO). System parameters, such as the number of neural nodes, rules, and learning coefficients are predefined. After initialization, new generations are produced through enhancement, crossover, and mutation operations.

2. Enhancement (PSO-Based Learning): After evaluating fitness, the top 50% of individuals are selected as elites. Instead of directly passing them to the next generation, they are first improved using PSO. In this step, the following occurs:

1. Elites are treated as a swarm;
2. Each individual updates itself based on
  (a) personal experience (cognitive learning);
  (b) best-performing individuals (social learning).

This process mimics natural learning, where individuals improve through both self-experience and interaction with others. It helps prevent premature convergence and enhances search capability. The improved elites form half of the next generation.

3. Crossover (GA-Based Evolution): The remaining population is generated using crossover among enhanced elites. Parents are selected using a tournament selection method, where individuals with better fitness are more likely to be chosen.

A two-point crossover technique is applied:

1. Two random crossover points are selected;
2. Genetic information between parents is exchanged;
3. Two new offspring are generated.

This strategy ensures that offspring inherit strong features from high-quality parents, improving overall population fitness. It also introduces diversity and supports global exploration. Figure 4 presents the flow chart of the Com_GAPSO_ process.

#### 2.1.3. Optimization Setup: Design Vector, Objective, and Constraints

The design vector is given by,

$$x=[W_{i},L_{i},I_{\mathrm{bias}},V_{\mathrm{bias}},C_{j},L_{k}]$$

where W_i_/L_i_ represent transistor dimensions of the transconductance and switching stages, I_bias_, *V*_bias_, denote bias voltages and currents, R_L_ and C_L_ are load components, and C_fb_ and C_var_ correspond to feedback and tunable capacitive elements used in the LBT and TCF-CS structures.

In our work, we consider the objective function in Equation (13),

(13) $$\max F=w_{1}\frac{CG}{CG_{\max}}+w_{2}\frac{IP_{1dB}}{IP_{1dB,\max}}-w_{3}\frac{P_{\mathrm{cons}}}{P_{\max}}-w_{4}\frac{NF}{NF_{\max}}$$

The objective function is designed to maximize conversion gain (CG) and linearity (represented by IP_1_dB) while penalizing power consumption and noise figure (NF). It should be noted that higher weights are assigned to CG and IP_1_dB compared to NF and power consumption.

**Figure 5:**
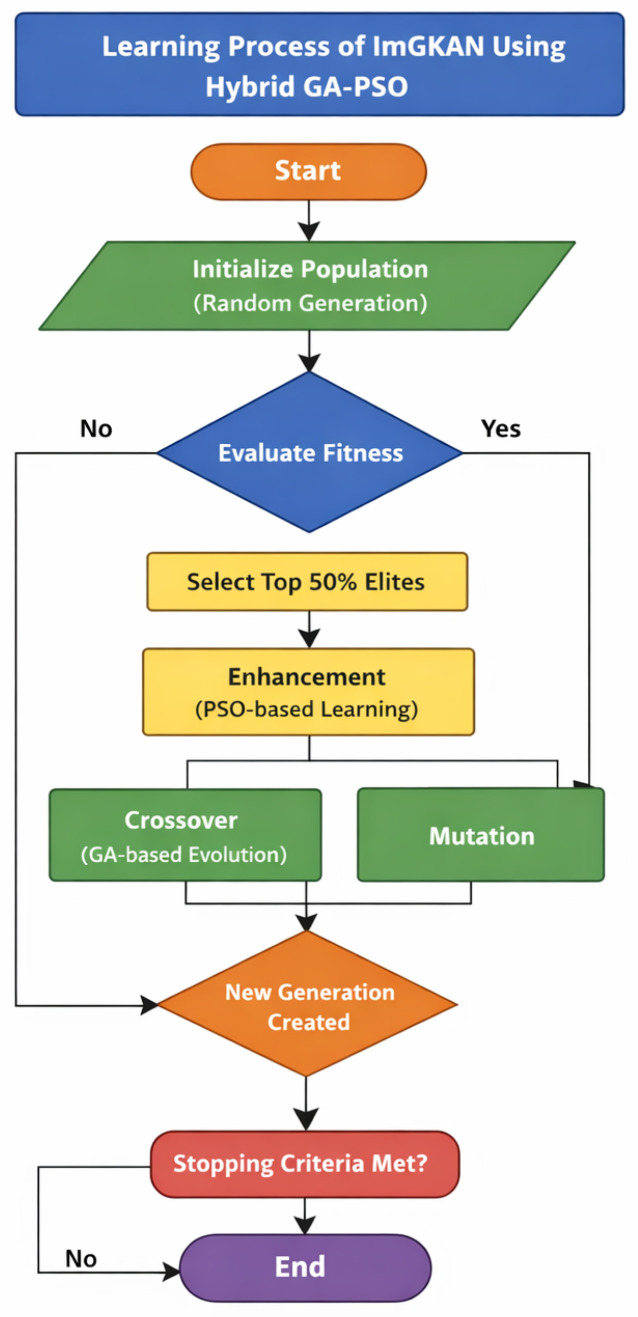
Algorithm learning process of Im_GKAN_ using ComGAPSO.

Figure 5 presents the algorithm learning process of Im_GKAN_ using the Com_GAPSO_ process. The figure illustrates the learning process of the Im_GKAN_ model using a hybrid GA and PSO approach. It begins with random initialization of the population, where candidate solutions are generated. Each solution is then evaluated based on a fitness function to determine its performance. The top 50% of the best-performing solutions are selected as elites for further improvement. These elite solutions undergo enhancement using PSO-based learning, followed by GA operations such as crossover and mutation to introduce diversity and explore new solutions. A new generation is created and the process repeats until the stopping criteria are satisfied, ensuring convergence to an optimal or near-optimal solution. Table 2 presents the parameter settings of the optimization algorithms.

#### 2.1.4. Testing Functions of Convergence Curve

In this section, we consider six benchmark functions, three of which are unimodal functions and three of which are multimodal functions. Equations (14) and (15) present the unimodal function and Equations (16)–(19) present the multimodal functions.

(14) $$\mathrm{func}1\left(x\right)=\sum_{i=1}^{n}x_{i}^{2}$$

(15) $$\mathrm{func}2\left(x\right)=\max_{i}\left\{|x_{i}|,\;1\leq i\leq n\right\}$$

(16) $$\mathrm{func}3\left(x\right)=\sum_{i=1}^{n-1}\left[100{\left(x_{i+1}-x_{i}^{2}\right)}^{2}+{\left(x_{i}-1\right)}^{2}\right]$$

(17) $$\mathrm{func}4\left(x\right)=\sum_{i=1}^{n}-x_{i}\sin\left(\sqrt{\left|x_{i}\right|}\right)$$

(18) $$\mathrm{func}5\left(x\right)=\sum_{i=1}^{n}\left[x_{i}^{2}-10\cos\left(2\pi x_{i}\right)+10\right]$$

(19) $$\mathrm{func}6\left(x\right)=-20\exp\left(-0.2\sqrt{\frac{1}{n}\sum_{i=1}^{n}x_{i}^{2}}\right)-\exp\left(\frac{1}{n}\sum_{i=1}^{n}\cos\left(2\pi x_{i}\right)\right)+20+e$$

**Figure 6:**
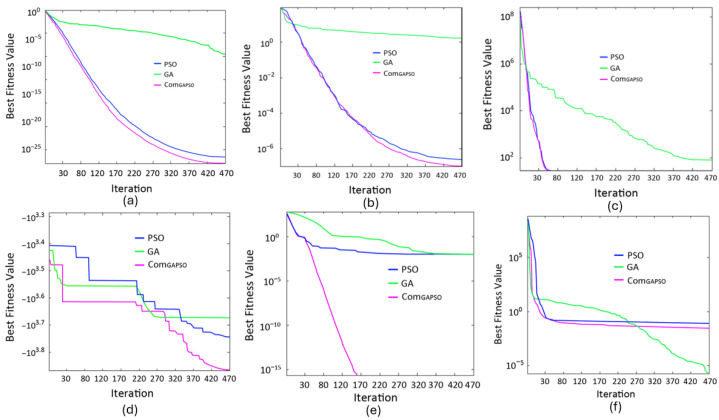
Convergence curve of GA, PSO, and Com_GAPSO_ variants on unimodal and multimodal functions (**a**) F1, (**b**) F2, (**c**) F3, (**d**) F4, (**e**) F5, (**f**) F6.

The convergence results in Figure 6 quantitatively demonstrate the superiority of the proposed Com_GAPSO_ algorithm over conventional GA and PSO methods across all benchmark functions. In unimodal cases (Figure 6a,b), Com_GAPSO_ achieves final fitness values on the order of 10^−26^ and 10^−7^, respectively, compared to PSO (10^−24^, 10^−6^) and GA (10^−6^, 10^0^, indicating an improvement of approximately 2–6 orders of magnitude over GA and 1–2 orders over PSO. For multimodal functions (Figure 6c–f), the proposed method consistently avoids premature convergence and reaches lower minima; for example, in Figure 6e, Com_GAPSO_ attains a fitness value near 10–15, significantly outperforming PSO 10^−7^ and GA 10^−0^, corresponding to improvements of 10 and 15 orders of magnitude, respectively. Similarly, in Figure 6d, Com_GAPSO_ achieves approximately 10^−3.9^, compared to PSO 10^−3.8^ and GA 10^−3.7^, showing steady incremental gains. In Figure 6f, although PSO converges rapidly initially, it stabilizes around 10^−1^, whereas Com_GAPSO_ maintains a lower level near 10^−2^, while GA has significantly slower convergence.

**Figure 7:**
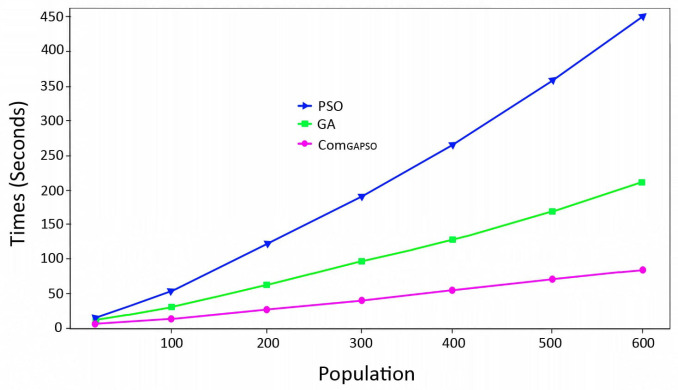
Computational cost of GA, PSO, and Com_GAPSO_ variants vs. Population.

Figure 7 illustrates the computational cost (in seconds) of PSO, GA, and the proposed Com_GAPSO_ algorithm as a function of population size. As the population increases from 50 to 600, all three algorithms exhibit a near-linear growth in computational time; however, the rate of increase differs significantly among them. For PSO, the computational time increases steeply from approximately 15 s at population 50 to about 430 s at population 600, indicating the highest computational burden. In contrast, GA shows moderate growth, rising from around 10 s to 200 s over the same population range.

The proposed Com_GAPSO_ algorithm consistently demonstrates the lowest computational cost, increasing from approximately 5 s at population 50 to only about 75 s at population 600. This represents a substantial improvement: at population 600, Com_GAPSO_ reduces computation time by approximately 82.5% compared to PSO and 62.5% compared to GA.

Even at mid-range population (e.g., 300), Com_GAPSO_ (35 s) is significantly faster than GA (90 s) and PSO (180 s), achieving roughly 80% and 61% reductions, respectively. Additionally, the slope of the Com_GAPSO_ curve is much lower, indicating better scalability and computational efficiency as population size increases.

#### 2.1.5. Relevance of Benchmark Optimization to Mixer Design

In the case of the unimodal benchmark functions, these evaluate the local exploitation capability of the optimization algorithm. Fast convergence on these functions indicates that Com_GAPSO_ can efficiently refine circuit parameters once a promising design region has been identified. In the mixer optimization process, this behavior is particularly important for accurately tuning transistor sizing, bias conditions, and matching-network elements to maximize conversion gain while maintaining acceptable noise and linearity performance.

The multimodal benchmark functions emulate the existence of multiple local optima commonly encountered in RF circuit design. For the proposed mixer, numerous combinations of device dimensions and bias conditions may satisfy a subset of performance requirements while degrading others. The superior performance of Com_GAPSO_ on multimodal functions demonstrates its ability to avoid premature convergence and locate globally optimal design solutions.

The computational-cost analysis highlights the practical advantage of the proposed optimization approach. Since each candidate mixer solution requires circuit-level evaluation through ADS simulations, optimization runtime is strongly dependent on the number of required evaluations. The reduced computational cost achieved by Com_GAPSO_ translates into fewer simulation iterations and shorter design cycles.

To further quantify this benefit, the optimized mixer achieved an improvement in simulated CG from 4.2 dB to 5.2 dB (an approximately 24% enhancement), while reducing power consumption from 4.9 mW to 3.4 mW (an approximately 31% reduction). Additionally, the measured OP_1_dB improved from 4.1 dBm to 5.1 dBm, demonstrating enhanced linearity. The improvements show that the proposed optimization approach is not only effective on benchmark problems but also leads to noticeable enhancements in the actual RF performance of the 24 GHz mixer design.

## 3. Results and Discussion

This section presents the performance evaluation of the proposed 24 GHz up-conversion mixer using the optimized algorithm and compares it with a conventional design and measured results. The analysis focuses on key metrics such as CG across a wide frequency range to demonstrate the effectiveness of the proposed approach. The comparison highlights the impact of optimization on improving gain, bandwidth, and overall stability, while also considering practical variations observed in measurements. In this work, a MATLAB (R2024a)–ADS (2024) co-simulation is used to automate the mixer optimization process. The Com_GAPSO_ and Im_GKAN_ algorithm in MATLAB generates candidate design parameters, which are passed to ADS via scripting to update the circuit. ADS then performs simulations (S-parameters, harmonic balance, and noise analysis) to evaluate key metrics such as conversion gain, linearity, noise figure, and power. The results are returned to MATLAB, where they are used to update the optimization process iteratively. This closed-loop approach enables accurate circuit evaluation while efficiently exploring the design space.

**Figure 8:**
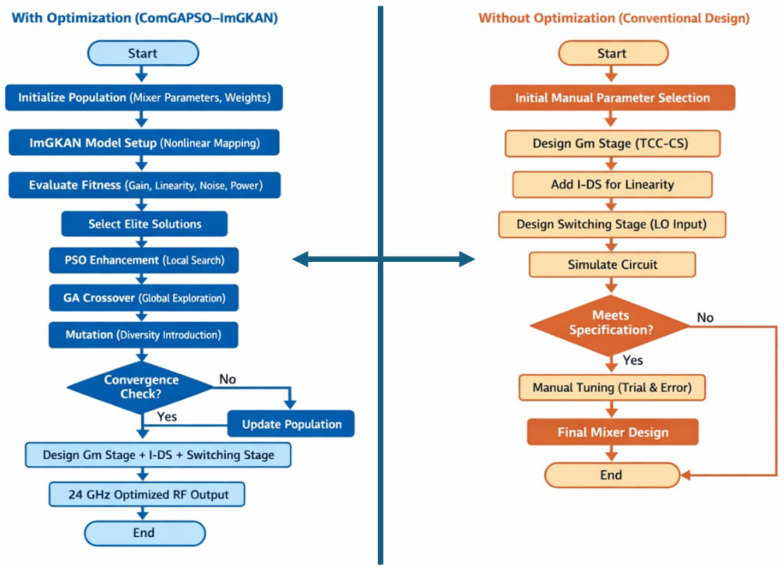
With optimization and without optimization process flow.

Figure 8 shows the process flow for circuit design with optimization and w/o optimization. The figure presents a comparative flow between an optimized and a conventional mixer design process. On the left, the optimized approach begins by initializing a population of mixer parameters and weights, followed by setting up the Im_GKAN_ model for nonlinear mapping. The system evaluates performance metrics such as gain, linearity, noise, and power, and then iteratively improves solutions using hybrid optimization techniques including PSO-based local search, GA crossover, and mutation. A convergence check determines whether the solution is optimal; if not, the population is updated and the process repeats. Once convergence is achieved, the design stages (Gm, I-DS, and switching stage) are finalized to produce a 24-GHz-optimized RF output.

In contrast, the conventional method on the right relies on manual parameter selection and sequential circuit design steps, including G_m_ stage design, linearity adjustments, and switching stage implementation. The circuit is simulated and repeatedly tuned through trial-and-error until the specifications are met, making the process more time-consuming and less efficient.

**Figure 9:**
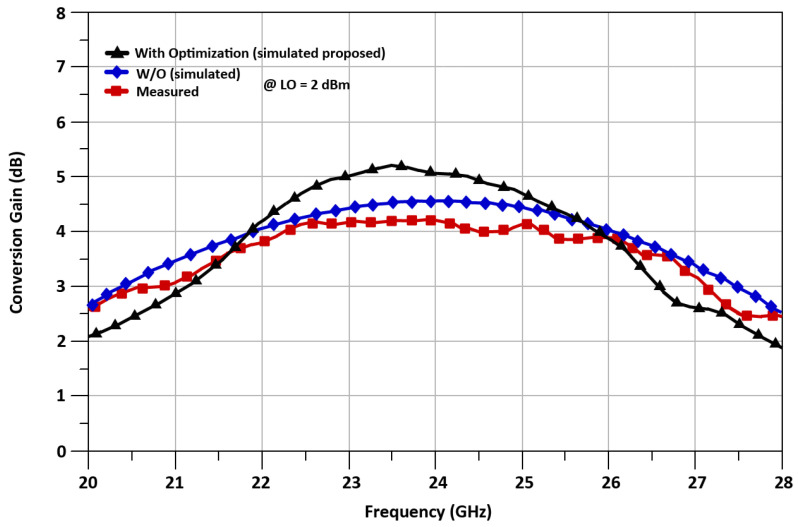
An analysis of the mixer’s CG with respect to frequency.

Figure 9 shows an analysis of the mixer’s CG with respect to frequency. This shows the CG performance of the proposed optimized mixer compared with the non-optimized and measured results over 20–28 GHz at an LO power of 2 dBm. The optimized design reaches a peak CG of about 5.2 dB near 24 GHz. The gain remains higher and more stable across the mid-band frequencies (22–25.5 GHz). The measured gain is 4.2 dB.

**Figure 10:**
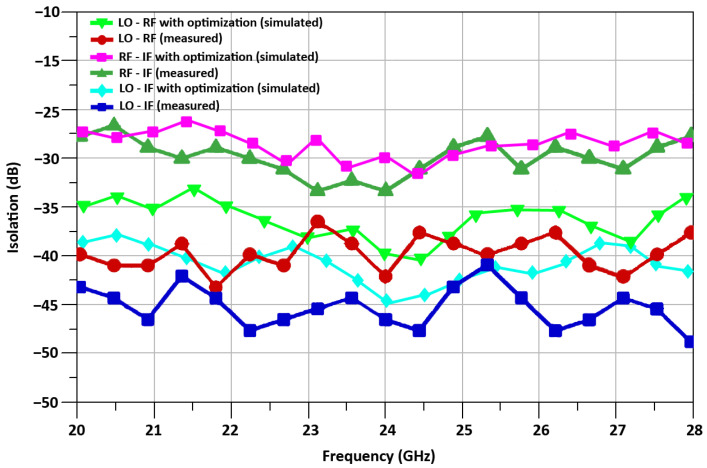
Mixer port isolation.

Figure 10 shows the mixer port isolation. In this case, a mixer (measured) with a 24 GHz bandwidth features an isolation between the LO-IF port, RF- IF port, and LO-RF port of −46.2 dB, −33.3 dB, and −42.4 dB, respectively. In the case of the optimized mixer simulation results, when we consider a frequency of 24 GHz, we see that the optimized up-conversion mixer demonstrates strong port-to-port isolation performance. Specifically, the LO-IF isolation reaches approximately −44 dB, indicating excellent suppression of local oscillator leakage into the IF port. The RF-IF isolation is around −30 dB, ensuring minimal undesired coupling between input and output paths, while the LO-RF isolation is maintained near −39 dB, reflecting efficient isolation between the LO and RF ports. A TCF-CS structure coupled with LBT, guided by the proposed optimization algorithm, successfully mitigates parasitic effects and nonlinear distortions.

**Figure 11:**
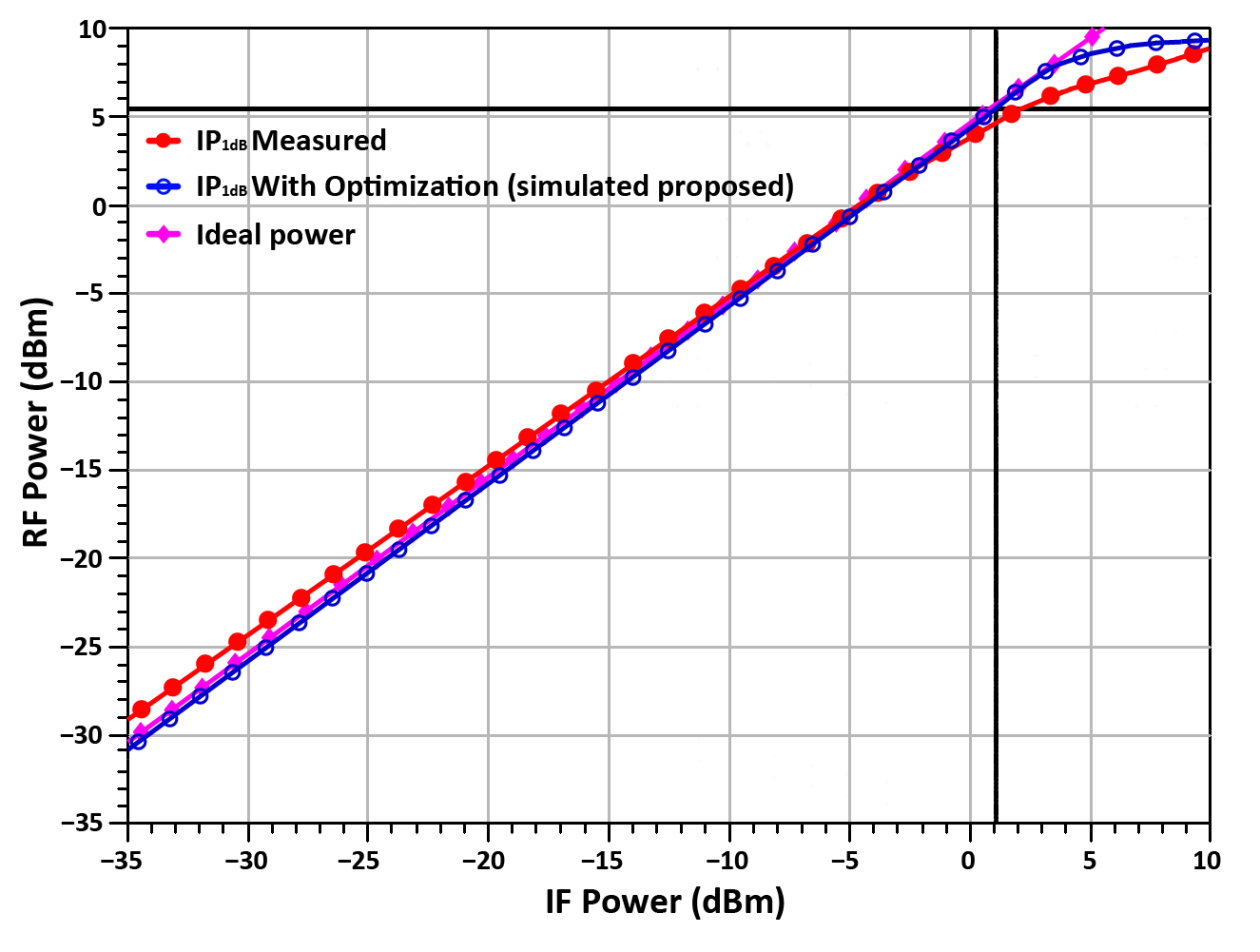
RF output power vs. IF input power.

As Figure 11 shows, the input 1-dB compression point characteristics of the proposed optimized up-conversion mixer demonstrate a significant improvement in linearity compared to the measured baseline. As illustrated in Figure 11, the optimized simulated results closely follow the ideal linear response over a wide input IF power range, indicating effective suppression of nonlinear distortion. The optimized mixer measured results shows a OP_1_dB of 5.1 dBm, and the IP_1_dB is −1.1 dBm, respectively, demonstrating good linear performance.

**Figure 12:**
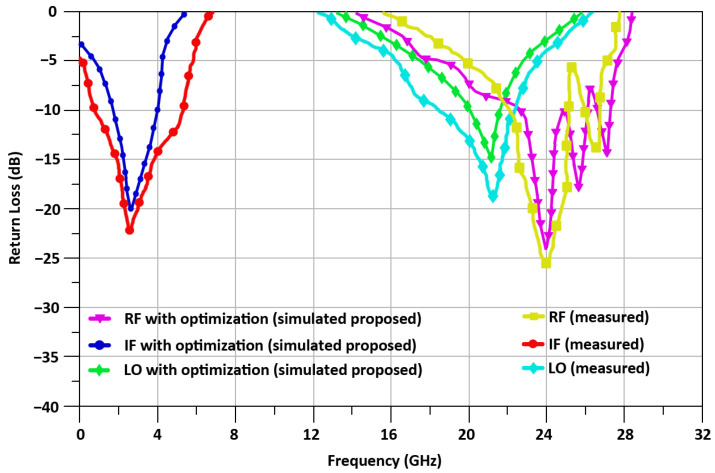
Return loss.

As can be seen in Figure 12, the proposed mixer has a measurable return loss. This mixer (measured) allows the IF port to be configured at 2.4 GHz with a return loss of −22.6 dB, the RF port to be configured at 24 GHz with a return loss of −26.1 dB, and the LO port to be configured at 21.6 GHz with a return loss of −19.1 dB. Furthermore in the case of optimized (simulated) design, the return loss of the IF, RF, and LO ports are −20 dB, −15.7 dB, and −24 dB, respectively. The RF port exhibits a deep return loss of approximately −24 dB near 24 GHz, indicating minimal signal reflection. Similarly, the LO port maintains a return loss of around −3 to −5 dB with improved matching observed across the operating band, while the IF port shows strong matching at lower frequencies with a minimum return loss reaching below −20 dB. Additionally, the LBT technique and the TCF-CS structure contribute further to the improvement in performance by compensating parasitic effects and stabilizing impedance characteristics in cooperation with each other. For B-5G applications operating in the 24 GHz band, the proposed mixer achieves reduced reflection losses, enhanced signal integrity, and improved overall RF performance.

**Figure 13:**
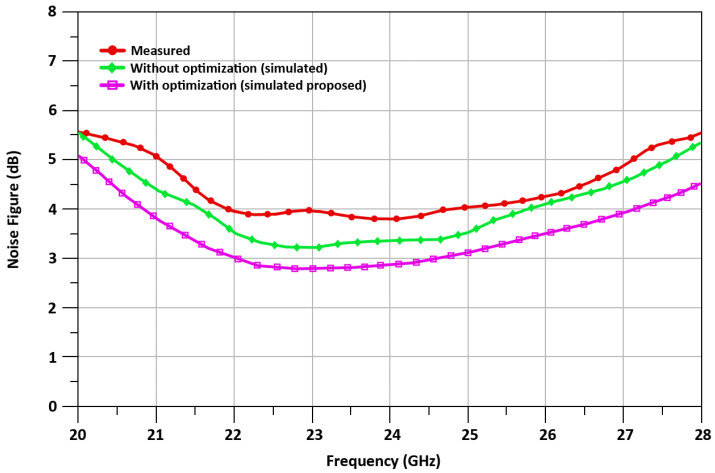
Noise Figure.

The noise figure (NF) performance is shown in Figure 13. This shows that the proposed optimized up-conversion mixer shows an improvement. As observed from the figure, the measured optimized design achieves a minimum NF of approximately 3.8 dB at 24 GHz.

### 3.1. Layout Considerations

**Figure 14:**
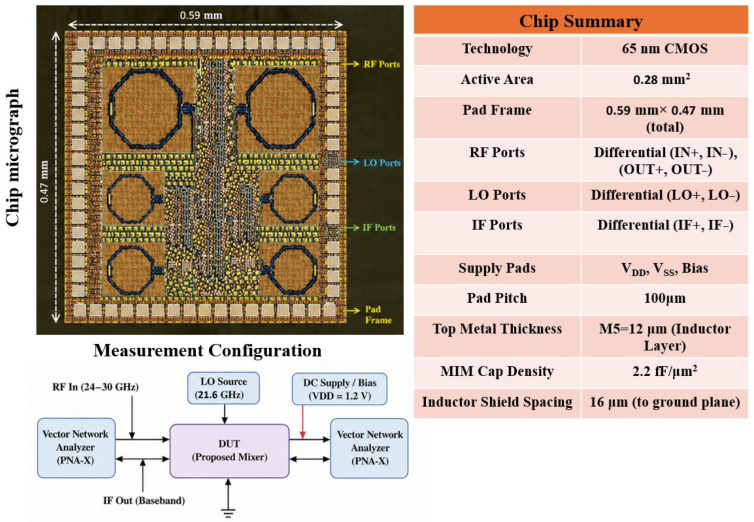
Chip micrograph.

The proposed mixer, incorporating the LBT and TCF-CS structures, is implemented using 65 nm CMOS technology. The chip micrograph is presented in Figure 14. The overall chip occupies an area of 0.28 mm^2^ (0.59 mm × 0.47 mm), excluding the pad region. The layout is carefully optimized to maintain stable circuit operation while minimizing parasitic resistances, capacitances, and inductances arising from interconnections, as well as diffusion-related parasitic capacitances.

To reduce gate resistance and parasitic capacitance, the transistors are segmented into multiple fingers. This multi-finger configuration also helps suppress nonlinearity caused by shunt capacitances, thereby improving gain performance. A multi-layer grounding strategy is adopted using several metal layers to create a low-resistance and low-inductance ground path. In addition, wide and thick power routing is implemented to ensure proper AC coupling to ground and to minimize voltage drops across the supply network.

Metal–insulator–metal (MIM) capacitors with a capacitance density of 2.2 fF/µm^2^ are employed in the design. The inductors are realized using the fifth metal layer of the 65 nm CMOS process, featuring a thickness of 12 µm and achieving a quality factor (Q) of approximately 12. To reduce electromagnetic interference (EMI) between inductors, a grounded shielding plane is placed at a distance of 16 µm from the inductor coils.

### 3.2. Measurement Setup and Validation

**Figure 15:**
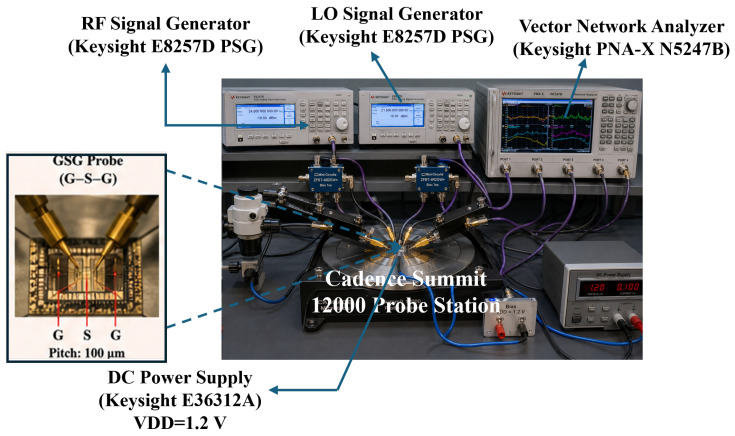
Measurement Setup.

The fabricated mixer was characterized through on-wafer measurements using Ground–Signal–Ground (GSG) RF probes. Figure 15 presents the measurement configuration. A Keysight PNA-X Vector Network Analyzer (VNA) was employed for RF characterization. The local oscillator (LO) signal was generated at 21.6 GHz and applied through the differential LO ports, while the RF input signal was swept from 24 GHz to 30 GHz. The device under test (DUT) was biased using a regulated DC power supply with VDD = 1.2 V through dedicated supply pads.

Prior to measurement, a standard Short–Open–Load–Thru (SOLT) calibration procedure was performed to establish the reference plane at the probe tips and eliminate the effects of cables, connectors, and external fixtures. The reported results therefore represent the intrinsic performance of the fabricated mixer. All measurements were conducted under room-temperature conditions using on-wafer probing without packaging. The measured conversion gain, port isolations, return losses, linearity metrics (IP_1_dB and OP_1_dB), and noise figure were obtained under these calibrated conditions, ensuring reliable validation of the proposed Com_GAPSO_–Im _GKAN_ optimized mixer design.

Table 3 shows the comprehensive performance comparison of 24 GHz CMOS up-conversion mixers, and Table 4 presents a comparison between prior work and this work.

### 3.3. Practical Application Prospects

While considering the practical application prospects, the proposed Com_GAPSO_–Im _GKAN_-optimized 24 GHz up-conversion mixer is fit for emerging Beyond-5G and future 6G wireless communication systems that require high CG, improved linearity, and low power consumption. The proposed optimization approach can be extended to the design of other RF front-end building blocks, including low-noise amplifiers, power amplifiers, and frequency synthesizers. From a system-level perspective, the enhanced CG improves signal quality and receiver sensitivity, while the improved linearity minimizes intermodulation distortion and unwanted spectral emissions. Such performance characteristics are critical in next-generation communication systems that require reliable operation under high data traffic, limited spectral resources, and complex interference conditions. The Com_GAPSO_–Im _GKAN_ approach significantly reduces the design space exploration time by intelligently identifying optimal transistor dimensions and biasing conditions, thereby shortening the RF integrated circuit development cycle. Consequently, the proposed methodology provides a scalable and efficient solution for future millimeter-wave RF front-end designs requiring simultaneous optimization of gain, linearity, power consumption, and overall circuit performance.

In addition, the proposed mixer architecture can be integrated with advanced intelligent sensing platforms that combine sensor-interface circuits and wireless communication modules. For example, smart sensing systems employing WO_3_/CNT-based sensors for gas detection and environmental monitoring can benefit from the proposed high-linearity and energy-efficient mixer to enable reliable wireless transmission of sensor data. Furthermore, its ability to efficiently handle multi-objective design constraints makes it attractive for automotive radar, intelligent sensing, IoT communication, and high-speed wireless transceiver applications operating in the millimeter-wave spectrum.

## 4. Conclusions

This paper presented a 24 GHz optimized CMOS up-conversion mixer for beyond-5G applications using a novel hybrid optimization framework based on Com_GAPSO_–Im _GKAN_. By combining the global exploration capability of GAs with the rapid convergence characteristics of PSO, the proposed approach enables precise and efficient parameter optimization for RF circuit design. The integration of the Im _GKAN_ model further enhances system performance through accurate nonlinear modeling and compensation. Moreover, the incorporation of the enhanced LBT together with the TCF-CS structure effectively suppresses third-order distortion and mitigates parasitic effects, contributing to improved overall mixer performance.

A peak CG of approximately 4.2 dB is achieved near 24 GHz. Furthermore, a mixer (measured) with a 24 GHz bandwidth features an isolation between the LO-IF port, RF-IF port, and LO-RF port of −46.2 dB, −33.3 dB, and - 42.4 dB, respectively. The isolation characteristics are LO–IF isolation of −44 dB, RF-IF isolation of −30 dB, and LO-RF isolation of −39 dB. The design achieves a measured OP_1dB_ of 5.1 dBm and IP_1dB_ of −1.1 dBm, demonstrating robust large-signal handling capability. Furthermore, impedance matching performance is also well maintained, with the RF port exhibiting a deep return loss of approximately −24 dB at 24 GHz. The LO port shows a return loss in the range of −3 to −5 dB, with improved matching across the operating band, while the IF port demonstrates strong low-frequency matching with return loss values below −20 dB. In addition, the proposed mixer achieves a measured minimum NF of 3.8 dB, indicating low noise degradation and suitability for high-frequency B-5G communication systems. Despite the promising results, the proposed framework can be further extended to higher frequency bands, such as 60 GHz and terahertz ranges, to support emerging 6G applications.

## Figures and Tables

**Table 1:** Proposed mixer simulation parameters (complete device-level details).

Components/Parameter	Dimension/Value	Components/Parameter	Dimension/Value
Transistor Dimensions (NMOS/PMOS)		Passive Elements	
*M*_1_, *M*_2_ (RF pair)	33 μm/65 nm	*L*_1_ − *L*_2_ (RF load)	172 pH
*M*_*p*_ (LO pair)	50 μm/65 nm	*L*_*s*1_ (LO matching)	70 pH
*M*_s_ (Switching pair)	40 μm/65 nm	*L*_*s*2_ (IF load)	90 pH
*M*_3_ − *M*_6_ (IF stage)	35 μm/65 nm	*C*_3_ − *C*_6_ (Coupling/Blocking)	43 fF
Varactor (Tuning Network)		Bias Conditions	
*C*_*v*1_ − *C*_*v*2_ (Varactors)	12–38 fF	*V*_*DD*_ (Supply Voltage)	1.2 V
Tuning Voltage (*V*_tune_)	0.5–1.2 V	*V*_bias,RF_ (*M*_1_–*M*_2_)	0.55 V
Q-Factor (at 24 GHz)	>18	*V*_bias,LO_ (*M*_*p*_)	0.60 V
		*V*_bias,IF_ (*M*_3_–*M*_6_)	0.50 V
Resistors and Biasing		Supply Current Distribution	
*R*_bias1_ (Gate bias)	1.5 k Ω	*I*_RF_ (*M*_1_–*M*_2_)	1.88 mW
*R*_bias2_ (Source degeneration)	200 Ω	*I*_LO_ (*M*_*p*_)	1.36 mW
*R*_load_ (IF termination)	100 Ω	*I*_IF_ (*M*_3_–*M*_6_)	0.98 mW
		Total Current (*I*_*DD*_)	3.4 mW
Output Matching Network		Simulation Settings	
$C_{\mathrm{RF\_match}}$ (Series)	15 fF	RF Frequency	24 GHz
$L_{\mathrm{RF\_match}}$ (Shunt)	160 pH	IF Frequency	2.4 GHz
$C_{\mathrm{IF\_match}}$ (Coupling)	20 fF	LO Drive Level	2 dBm
$R_{\mathrm{IF\_term}}$ (IF Load)	100 Ω	Verification Method	Simulation, Post-Layout Simulation, and Measurement

**Table 2:** Parameter Settings.

Category	Parameter	Symbol	Value (Used)	Description
Optimization	Population Size	–	50	Number of individuals
	Number of Generations	–	100	Iteration count
	Crossover Rate	*P*_*c*_	0.8	GA crossover probability
	Mutation Rate	*P_m_*	0.05	GA mutation probability
	Inertia Weight	*w*	0.7	PSO exploration factor
	Cognitive Coefficient	*c*_1_	1.5	Personal learning factor
	Social Coefficient	*c*_2_	1.5	Global learning factor
Im_GKAN_ Model	Number of Nodes	–	15	Hidden structure size
	Learning Rate	–	0.01	Training step size
	Activation Function	–	Adaptive spline	Nonlinear mapping
	Training Method	–	Com_GAPSO_-Hybrid optimization based	Surrogate-assisted hybrid optimization
Design and Evaluation	Design Vector	–	[*W*_i_, *L*_i_, *I*_bias_, *V*_bias_, *C*_*j*_, *L*_*k*_]	Circuit design variables
	Objective Function Weights	–	[0.30, 0.20, 0.20, 0.20]	For [CG, NF, *IP*_1dB_, Power]
	RF Constraints	–	$\begin{array}{cc} & CG\geq 5\;\mathrm{dB},\;NF\leq 3.8\;\mathrm{dB},\\ & IP_{1\mathrm{dB}}\geq -1\;\mathrm{dBm},\\ & \mathrm{Isolation}\leq -30\;\mathrm{dB},\\ & S_{11}\leq -10\;\mathrm{dB} \end{array}$	Performance specifications
	Simulator	–	ADS/Cadence Spectre RF	Circuit-level evaluation

**Table 3:** Comparison with recently reported up-conversion mixers.

Ref.	Year	Proc.	RF Freq.	Area	RF–IF	LO–IF	LO–RF	RF RL	LO RL	IF RL	Gain	OP1dB	NF	Power
		**(nm)**	**(GHz)**	**(mm^2^)**	**(dB)**	**(dB)**	**(dB)**	**(dB)**	**(dB)**	**(dB)**	**(dB)**	**(dBm)**	**(dB)**	**(mW)**
Won et al. [27]	2015	130	23.4–29.2	0.858	68.9	N/R	28.9	>10	>10	>10	−1.9	0.3	N/R	22.8
Siddique et al. [28]	2021	65	24	0.40	40.0	27.3	35.1	−20.7	−24.8	−22.6	4.7	0.41	3.8	5.2
Delwar et al. [29]	2022	65	24	0.42	18.1	24.3	22.4	−21.5	−24.7	−22.45	3.5–4.0	∼1.0	∼4.0	∼6.0
Chen et al. [30]	2025	180	2–18	1.20	N/R	44–65	22–35	<−8	N/R	N/R	10–16	N/R	N/R	3.69
**This Work**	**2026**	**65**	**24**	**0.28**	**−30**	**−44**	**−39**	**−24.1**	**−19.1**	**−22.6**	**4.2**	**5.1**	**3.8**	**3.4**

**Table 4:** Performance comparison of prior work and proposed work.

Parameter	Prior Work (Without Optimization)		This Work (With Optimization)	
	**Simulated**	**Measured**	**Simulated**	**Measured**
Gain (dB)	4.2	3.9–4.0	5.2	4.2
NF (dB)	3.5	3.8	2.9	3.8
OP_1dB_ (dBm)	4.5	4.1	6.0	5.1
P_1dB_ (dBm)	0	0.67	0	−1.1
Chip Area (mm^2^)	–	0.40	–	0.28
Power Consumption (mW)	–	4.9	–	3.4

## Data Availability

The data presented in this study are available from the corresponding author upon reasonable request.
